# Maternal lipid profile in early pregnancy is associated with foetal growth and the risk of a child born large-for-gestational age: a population-based prospective cohort study: Maternal lipid profile in early pregnancy and foetal growth

**DOI:** 10.1186/s12916-020-01730-7

**Published:** 2020-10-02

**Authors:** Maria C. Adank, Laura Benschop, Alet W. Kors, Kelly R. Peterbroers, Anna M. Smak Gregoor, Monique T. Mulder, Sarah Schalekamp-Timmermans, Jeanine E. Roeters Van Lennep, Eric A. P. Steegers

**Affiliations:** 1 Department of Obstetrics and Gynaecology, Erasmus MC, University Medical Centre, Rotterdam, the Netherlands; 2 Generation R Study Group, Erasmus MC, University Medical Centre, Rotterdam, the Netherlands; 3 Department of General Medicine, Erasmus MC, University Medical Centre, Rotterdam, the Netherlands

**Keywords:** Pregnancy, Lipoproteins, Foetal weight, Infant; Small-for-gestational age, Foetal programming

## Abstract

**Background:**

Lipids such as cholesterol and triglycerides play an important role in both maternal and foetal energy metabolism. Little is known about maternal lipid levels in pregnancy and their effect on foetal growth. The aim of this study was to assess maternal lipid levels, foetal growth and the risk of small-for-gestational age (SGA) and large-for-gestational age (LGA).

**Methods:**

We included 5702 women from the Generation R Study, a prospective population-based cohort. Maternal lipid levels (total cholesterol, triglycerides and high-density lipoprotein cholesterol [HDL-c]) were measured in early pregnancy (median 13.4 weeks, 90% range [10.5 to 17.2]). Low-density lipoprotein cholesterol (LDL-c), remnant cholesterol and non-HDL-c were calculated. Foetal growth was measured repeatedly by ultrasound. Information on birth anthropometrics was retrieved from medical records. A birth weight below the 10th percentile was defined as SGA and above the 90th percentile as LGA.

**Results:**

Maternal triglyceride and remnant cholesterol levels were associated with increased foetal head circumference and abdominal circumference growth rates. Triglycerides and remnant cholesterol were positively associated with the risk of LGA (odds ratio [OR] 1.11, 95% confidence interval [CI] [1.01 to 1.22] and OR 1.11, 95% CI [1.01 to 1.23], respectively). These associations were independent of maternal pre-pregnancy body mass index, but not maternal glucose levels. We observed no association between maternal lipids in early pregnancy and SGA.

**Conclusions:**

Our study suggests a novel association of early pregnancy triglyceride and remnant cholesterol levels with foetal growth, patterns of foetal growth and the risk of LGA. Future studies are warranted to explore clinical implication possibilities.

## Background

The worldwide incidence of overweight and obese women of reproductive age is increasing [1–3]. High maternal weight and hyperglycaemia are established risk factors for increased foetal growth and a child born large-for-gestational age (LGA). Maternal hyperglycaemia is associated with a higher flux of glucose over the placenta leading to foetal upregulation of insulin, increased foetal growth and ultimately a child born LGA [4–10].

Because the foetus has a limited capacity for de novo lipogenesis and fatty acid oxidation, it is dependent on maternal triglycerides as source for growth and development [11, 12]. During pregnancy, maternal insulin resistance leads to decreased lipoprotein lipase (LPL) activity and hence 2–3-fold increased maternal triglyceride levels [7, 8, 13]. Maternal triglycerides in the form of both liver-derived very low-density lipoprotein (VLDL) and dietary chylomicrons must first be hydrolysed to free fatty acids (FFA) by placental lipases to allow uptake by the syncytiotrophoblast [13, 14]. There FFA can be stored, metabolized, oxidized or transported into the foetal circulation [13, 15]. Insufficient fatty acid oxidation has been linked to preterm birth and intrauterine growth restriction [16]. On the other hand, maternal triglycerides were shown to correlate more strongly than glucose with newborn percent fat [17, 18].

Adverse birth outcomes, including small-for-gestational age (SGA) and LGA, may affect short-term (e.g. increased morbidity and mortality) and long-term (increased risk of hypertension, diabetes and metabolic syndrome) health of the child [19–21].

Lipid levels in early pregnancy are associated with maternal pregnancy complications, such as pre-eclampsia, independent of pre-pregnancy body mass index (BMI) [22]. Our hypothesis is that according to the Developmental Origins of Health and Disease (DOHaD) theory, maternal lipid levels may also lead to adverse birth outcomes such as LGA due to adverse growth patterns [23]. Therefore, the aim of this study is to examine the association of maternal early pregnancy lipid levels with foetal growth, patterns of foetal growth and the risk of SGA and LGA independent of maternal BMI and glucose levels.

## Methods

### Study design

**Fig. 1:**
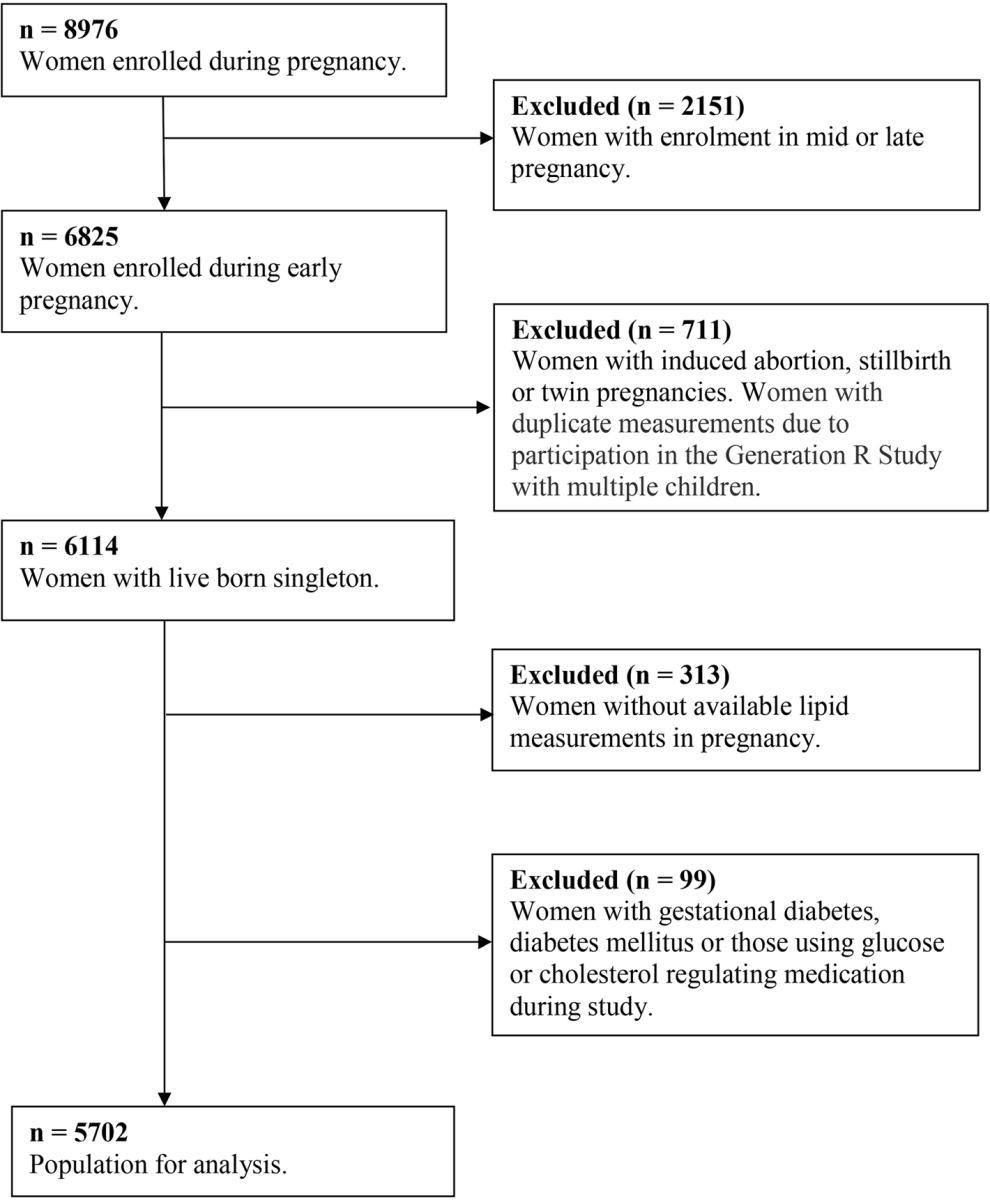
Flowchart showing the inclusion and exclusion criteria

This study was embedded within the Generation R Study, an ongoing population-based prospective cohort study from early pregnancy onwards in Rotterdam, the Netherlands [24]. All pregnant women living in Rotterdam with an expected delivery date between April 2002 and January 2006 were eligible for participation. The study has been approved by the Medical Ethical Committee of the Erasmus Medical Centre Rotterdam, the Netherlands (MEC 198.782/2001/31). All procedures were in accordance with institutional guidelines, and written informed consent was obtained from all participants [25]. For the present study, we included 5702 women with a live born singleton and available information on lipid measurements in early pregnancy. We excluded women with a twin pregnancy, diabetes mellitus and gestational diabetes and those on lipid or glucose regulating treatment during study enrolment (Fig. 1). Gestational diabetes was diagnosed according to Dutch guidelines using the following criteria: either a random glucose level > 11.0 mmol/L, a fasting glucose level ≥ 7.0 mmol/L or a fasting glucose level between 6.1 and 6.9 mmol/L with a subsequent abnormal glucose tolerance test (glucose level > 7.8 mmol/L after glucose intake) [24]. Additional file 2 contains a Strengthening the Reporting of Observational Studies in Epidemiology (STROBE) statement for the current study [26].

### Exposure: maternal lipid levels in early pregnancy

Non-fasting plasma samples were obtained in early pregnancy (median 13.4 weeks of gestation, 90% range [10.5 to 17.2]). Total cholesterol (mmol/L), triglyceride (mmol/L) and high-density lipoprotein cholesterol (HDL-c) (mmol/L) concentrations were analysed. LDL-c (mmol/L), remnant cholesterol ([total cholesterol − LDL-c] − HDL-c) and non-HDL-c (total cholesterol − HDL-c) were calculated [22]. Sample processing, storage procedures and the changes in maternal lipid levels per gestational age in the same study population have been described previously [22, 27].

### Outcome measures: foetal growth parameters and adverse birth outcomes

The primary outcome of this study was adverse birth outcomes. Secondary outcomes were foetal growth and foetal growth patterns. Ultrasound measurements were performed in mid-pregnancy (median 20.4 weeks of gestation, 90% range [19.1 to 22.5]) and late pregnancy (median 30.2 weeks of gestation, 90% range [29.1 to 31.9]) using protocols describing standardized planes [28–30]. Foetal growth parameters included the head circumference, femur length and abdominal circumference. We calculated the estimated foetal weight (EFW) using the Hadlock 3 formula [31]. Longitudinal growth curves and gestational-age-adjusted standard deviation scores (SDS) were constructed for all foetal growth parameters. These gestational-age-adjusted SDS were based on reference growth curves from the whole-study population and represent the equivalent of *z*-scores [30]. Information on birth anthropometrics (head circumference, length and weight) and gestational age at birth was obtained from medical files and midwife registries. Gestational-age-adjusted SDS for birth weight were constructed using North European growth standards as the reference growth curve and represent the equivalent of *z*-scores [32]. A birth weight below the 10th percentile, adjusted for gestational age and foetal sex, was defined as SGA and above the 90th percentile as LGA. All children with a birth weight above the 10th percentile and below the 90th percentile were considered average-for-gestational age (AGA).

### Covariates

Potential confounders were identified based on the graphical criteria for confounding by visualizing a directed acyclic graph (DAG) (Additional file 1, Figure S1). Of these potential confounders, we included those in the models that changed the effect estimates > 10% for at least one of the outcomes [33]. Maternal questionnaires at study enrolment provided information on maternal age, parity, ethnicity, education, pre-pregnancy body mass index, smoking habits and folic acid supplementation. Pre-pregnancy BMI was highly correlated with BMI measured at study enrolment (Pearson’s correlation coefficient 0.95 [*P* value < .001]) [34], and therefore, pre-pregnancy BMI was used in the analyses. Non-fasting maternal glucose levels were measured in early pregnancy with the c702 module on a Cobas 8000 analyser as described previously [6, 27]. Women who experienced gestational hypertension and pre-eclampsia in their index pregnancy were classified as having a hypertensive disorder of pregnancy. All diagnoses were cross-validated retrospectively by obstetric records that were obtained from midwife and hospital registries [24, 35]. The criteria for hypertensive disorders of pregnancy were defined by the criteria that applied at the time of study inclusion of Generation R. This was according to the statement from the International Society for the Study of Hypertension in Pregnancy of 2001 [36]. Several overlapping sources (obstetric caregivers and Municipal Health Services) provided information on admission to the Neonatal Intensive Care Unit (NICU) [37].

### Statistical analyses

Missing values of the covariates were imputed through multiple imputation procedures [38]. Data were imputed according to the Markov Chain Monte Carlo method. Data were analysed in each set separately, and pooled estimates from the five imputed datasets were used to report the effect estimates and their 95% confidence intervals. For the multiple imputation procedure, we performed 10 iterations [39]. In this study, 0% of women had missing information on maternal age at enrolment and gestational age at the time of blood sampling, 4.2% on ethnicity, 7.4% on educational level, 0.9% on parity, 10.4% on smoking habits, 23.2% on folic acid supplementation, 21.4% on pre-pregnancy BMI and 2.4% on early pregnancy maternal glucose levels. First, we examined baseline characteristics. Thereafter, we examined differences in maternal lipid profiles in women with a child born AGA compared to women with a child born SGA or LGA through a Student *t* test. Not normally distributed exposure measures were log transformed. To enable comparison of effect estimates, we constructed SD scores (SDS) of exposures. Multivariate linear regression analyses were performed to examine the associations of maternal lipid levels in early pregnancy with foetal growth parameters at birth. We performed multivariate logistic regression analyses to determine the association of lipid levels in early pregnancy with the risk of SGA and LGA. The basic regression model adjusted for maternal age at enrolment, gestational age at the time of blood sampling, parity, ethnicity, educational level, smoking habits and folic acid supplementation. The BMI model additionally adjusted for pre-pregnancy BMI. In a separate glucose model, we additionally adjusted for early pregnancy maternal glucose levels. To assess foetal growth patterns, we examined the associations between lipid levels in early pregnancy and repeatedly measured foetal growth parameters using unbalanced repeated measurement regression models with an unstructured covariance structure. These models take the correlation between repeated measurements of the same individual into account and allow for incomplete outcome data [40]. We included maternal early pregnancy lipid levels in these models as an intercept and as an interaction term with gestational age to estimate foetal growth rates over time [40]. The repeated analyses were conducted without adjustment for covariates, which most clearly reflects clinical practice. Since maternal lipid levels in early pregnancy are associated with pre-eclampsia and since hypertensive disorders of pregnancy are associated with adverse birth outcomes, we additionally performed a regression analysis of lipid levels in early pregnancy with the risk of SGA in LGA in a subgroup excluding women with gestational hypertension or pre-eclampsia in their index pregnancy.

To examine whether the association of early pregnancy lipid levels with adverse birth outcomes was explained by genetic factors and/or lifestyle factors as well, we performed logistic regression analyses in a subset of women including only nulliparous, non-smoking women with a pre-pregnancy BMI < 25 kg/m^2^. Comparison of women included and excluded in this study was conducted by comparing the characteristics of women included in this study (women with inclusion in early pregnancy, [*n* = 5702]) to women with inclusion in mid- or late pregnancy (*n* = 2151).

In all analyses, a *P* value < .05 was considered statistically significant. Statistical analyses were performed using the IBM Statistical Package for the Social Sciences version 24.0 for Windows (SPSS Incl., Chicago, IL, USA) and the Statistical Analysis System version 9.4 (SAS, Institute Inc., Cary, NC, USA).

## Results

We included 5702 women. These women were on average 29.5 (± 5.1) years of age, mostly European (58.6%), and most women had a pre-pregnancy BMI < 25.0 kg/m^2^ (71.6%). Foetal growth parameters were available in 5499 (96.4%) and 5486 (96.2%) children in mid- and late pregnancy, respectively (Table 1). Of all children, 4526 were born AGA, 564 SGA and 565 LGA. Women with a child born LGA had higher levels of triglycerides and remnant cholesterol in early pregnancy than women with a child born AGA (Table 2). No differences were observed in lipid distribution between women with a child born SGA and AGA.

**Table 1:** Baseline characteristics (*n* = 5702)

Outcomes	
**Maternal characteristics**	
Maternal age at enrolment (years)	29.5 (5.1)
Non-European ethnicity, *n* (%)	2362 (41.4)
Educational level	
Primary or no education, *n* (%)	667 (11.7)
Secondary, *n* (%)	2644 (46.4)
Higher, *n* (%)	2391 (41.9)
Pre-pregnancy BMI (kg/m^2^)	
Normal or underweight (< 25.0), *n* (%)	4081 (71.6)
Overweight (25.0–30.0), *n* (%)	1143 (20.0)
Obesity (≥ 30.0), *n* (%)	478 (8.4)
Nulliparous, *n* (%)	3506 (61.5)
Smoking during pregnancy, *n* (%)	1642 (28.8)
No folic acid supplementation, *n* (%)	1609 (28.2)
Gestational age at blood sampling (weeks)	13.4 (10.5 to 17.2)
Glucose levels (mmol/L)	4.4 (0.8)
**Foetal characteristics**	
Mid-pregnancy measurements, *n* (%)	5499 (96.4)
Gestational age (weeks)	20.5 (19.1 to 22.6)
Head circumference (mm)	179 (13)
Femur length (mm)	33 (3)
Abdominal circumference (mm)	156 (14)
Estimated foetal weight (g)	372 (77)
Late pregnancy measurements, *n* (%)	5486 (96.2)
Gestational age (weeks)	30.4 (29.0 to 32.2)
Head circumference (mm)	285 (12)
Femur length (mm)	57 (3)
Abdominal circumference (mm)	263 (16)
Estimated foetal weight (g)	1604 (177)
**Birth characteristics**	
Gestational age at birth (weeks)	40.1 (36.9 to 42.1)
Boy, *n* (%)	2880 (50.5)
Birth measurements, *n* (%)	5666 (99.4)
Head circumference (mm)	338 (17)
Length (mm)	502 (24)
Birth weight (g)	3401 (560)
Gestational hypertension, *n* (%)	218 (3.8)
Pre-eclampsia, *n* (%)	139 (2.4)
Early onset (< 34 weeks of gestation), *n*	18
Late onset (> 34 weeks of gestation), *n*	121
NICU admission	595 (10.4)

*Abbreviation*: *BMI* body mass index. Data are presented as valid percentages for categorical variables, mean (SD) for continuous variables with a normal distribution or median (90% range) for continuous variables with a skewed distribution. Covariates are imputed

**Table 2:** Maternal lipid profile in early pregnancy and foetal growth

	SGA (*n* = 564)	AGA (*n* = 4526)	LGA (*n* = 565)
Gestational age at blood sampling, weeks	13.4 (10.5 to 17.2)	13.4 (10.5 to 17.2)	13.2 (10.9 to 17.1)
Total cholesterol, mmol/L	4.77 (0.90)	4.82 (0.87)	4.83 (0.88)
Triglycerides, mmol/L	1.23 (0.68 to 2.33)	1.26 (0.72 to 2.34)	1.33 (0.73 to 2.51)^a^
LDL-c, mmol/L	2.40 (0.74)	2.43 (0.72)	2.43 (0.73)
HDL-c, mmol/L	1.77 (0.35)	1.78 (0.35)	1.74 (0.35)^a^
Remnant cholesterol, mmol/L	0.56 (0.31 to 1.06)	0.57 (0.33 to 1.06)	0.60 (0.33 to 1.12)^a^
Non-HDL-c, mmol/L	3.00 (0.85)	3.05 (0.83)	3.08 (0.85)

*Abbreviations*: *SGA* small-for-gestational age, *AGA* average-for-gestational age, *LGA* large-for-gestational age. *LDL-c* low-density lipoprotein cholesterol, *HDL-c* high-density lipoprotein cholesterol. Data are presented as mean (SD) for continuous variables with a normal distribution, or as median (90% range) for continuous variables with a skewed distribution

^a^Student *t* test value of *P* < 0.05 between women with LGA and women with AGA

**Fig. 2:**
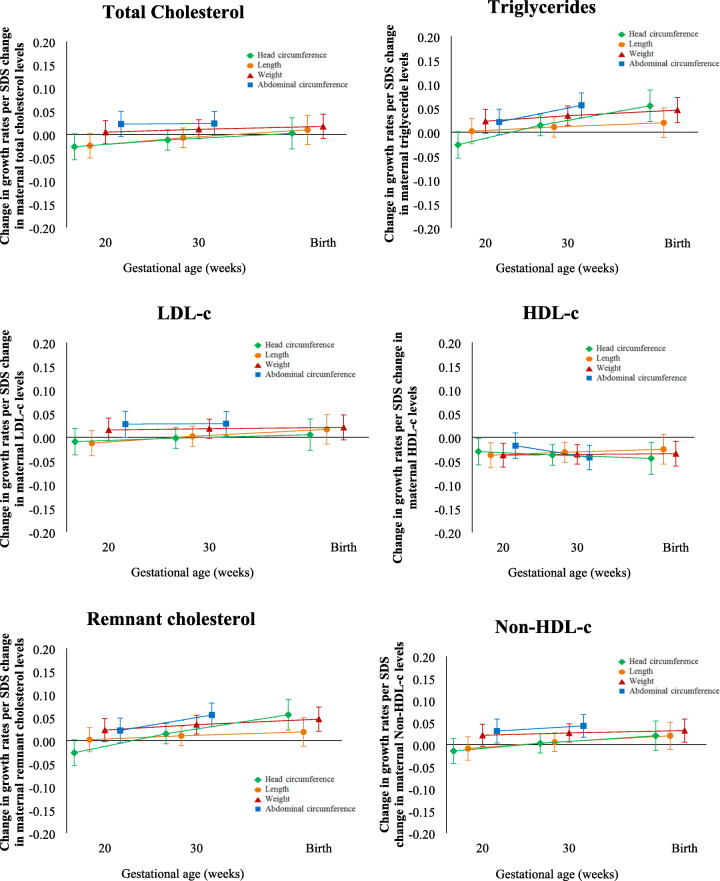
Differences in foetal growth rates per change in maternal early pregnancy lipid level. *Abbreviations*: SDS, standard deviation score; CI, confidence interval; LDL-c, low-density lipoprotein cholesterol; HDL-c, high-density lipoprotein cholesterol. Estimates represent SDS values (95% CI) from repeated measurement regression models that reflect the differences in gestational age-adjusted growth rates in SDS of head circumference, length, weight and abdominal circumference at mid-pregnancy, late pregnancy and at birth per 1 SDS change in maternal early pregnancy lipid levels

Figure 2 shows that triglycerides and remnant cholesterol were associated with increased foetal head circumference growth rates from late pregnancy onwards resulting in a higher head circumference at birth (0.6 SDS, 95% confidence interval [CI] [0.04 to 1.2] and 0.7 SDS, 95% CI [0.1 to 1.2], respectively) (Table 3). Additionally, triglycerides and remnant cholesterol were associated with increased foetal abdominal circumference growth rates from mid-pregnancy onwards (Fig. 2) resulting in a higher birth weight (18.4 SDS, 95% CI [6.5 to 30.3] and 18.7 SDS 95% CI [6.8 to 30.6], respectively) (Table 3). Maternal lipid levels in early pregnancy were not associated with foetal length and weight growth patterns (Fig. 2 and Table 3).

**Table 3:** Associations of maternal lipid levels in early pregnancy with birth anthropometrics

	Head circumference (mm) *β* (95% CI) ***n*** = 3110	***P*** value	Birth length (mm) *β* (95% CI) ***n*** = 3641	***P*** value	Birth weight (g) *β* (95% CI) ***n*** = 5666	***P*** value
**Total cholesterol, SDS**						
Basic model	0.2 (− 0.4 to 0.8)	0.44	0.1 (− 0.6 to 0.9)	0.73	9.2 (− 2.5 to 20.9)	0.12
BMI model	0.1 (− 0.4 to 0.7)	0.65	− 0.1 (− 0.8 to 0.7)	0.89	3.2 (− 8.4 to 14.8)	0.59
Glucose model	0.1 (− 0.4 to 0.7)	0.63	− 0.0 (− 0.8 to 0.7)	0.90	3.6 (− 8.1 to 15.2)	0.55
**Triglycerides, SDS**						
Basic model	0.8 (0.2 to 1.3)	0.01	0.6 (− 0.2 to 1.3)	0.13	29.6 (17.9 to 41.3)	< 0.001
BMI model	0.6 (0.1 to 1.2)	0.03	0.3 (− 0.5 to 1.0)	0.48	19.5 (7.6 to 31.3)	0.001
Glucose model	0.6 (0.04 to 1.2)	0.04	0.2 (− 0.5 to 1.0)	0.53	18.4 (6.5 to 30.3)	0.002
**LDL-c, SDS**						
Basic model	0.2 (−0.4 to 0.8)	0.51	0.2 (− 0.5 to 1.0)	0.51	10.2 (− 1.3 to 21.7)	0.08
BMI model	0.1 (− 0.5 to 0.7)	0.79	0.0 (− 0.7 to 0.7)	0.96	2.9 (− 8.5 to 14.4)	0.62
Glucose model	0.1 (− 0.5 to 0.7)	0.78	0.0 (− 0.7 to 0.7)	0.95	3.2 (− 8.3 to 14.7)	0.58
**HDL-c, SDS**						
Basic model	− 0.4 (− 1.0 to 0.2)	0.15	− 0.7 (− 1.4 to 0.1)	0.07	−20.1 (−31.7 to − 8.5)	0.001
BMI model	− 0.3 (− 0.9 to 0.3)	0.27	− 0.4 (− 1.2 to 0.3)	0.22	−12.7 (− 24.3 to − 1.1)	0.03
Glucose model	− 0.3 (− 0.9 to 0.3)	0.32	− 0.4 (− 1.2 to 0.3)	0.25	−11.6 (− 23.2 to 0.1)	0.05
**Remnant cholesterol, SDS**						
Basic model	0.8 (0.2 to 1.4)	0.01	0.6 (− 0.1 to 1.3)	0.11	30.0 (18.3 to 41.8)	< 0.001
BMI model	0.7 (0.1 to 1.3)	0.02	0.3 (− 0.4 to 1.0)	0.44	19.8 (7.9 to 31.6)	0.001
Glucose model	0.7 (0.1 to 1.2)	0.03	0.3 (− 0.5 to 1.0)	0.48	18.7 (6.8 to 30.6)	0.002
**Non-HDL-c, SDS**						
Basic model	0.4 (− 0.2 to 1.0)	0.18	0.4 (− 0.3 to 1.1)	0.27	17.7 (6.1 to 29.3)	0.003
BMI model	0.3 (− 0.3 to 0.8)	0.38	0.1 (− 0.6 to 0.9)	0.74	8.4 (− 3.3 to 20.0)	0.16
Glucose model	0.3 (− 0.3 to 0.8)	0.39	0.1 (− 0.6 to 0.8)	0.74	8.2 (− 3.4 to 19.9)	0.17

*Abbreviations*: *CI* confidence interval, *SDS* SD scores, *LDL-c* low-density lipoprotein cholesterol, *HDL-c* high-density lipoprotein cholesterol. Values are beta’s (95% CI) derived from multiple linear regression analyses. Basic model: adjusted for maternal age, gestational age at blood sampling, ethnicity, educational level, parity, smoking, folic acid supplementation and gestational age at measurement. BMI model: basic model additionally adjusted for pre-pregnancy BMI. Glucose model: BMI model additionally adjusted for glucose levels in early pregnancy

Table 4 shows the association of maternal lipid concentrations in early pregnancy with adverse birth outcomes. Triglyceride and remnant cholesterol levels in early pregnancy were positively associated with the risk of LGA. The association of triglycerides and remnant cholesterol with LGA was attenuated when adjusting for pre-pregnancy BMI. However, after adjustment for glucose, the associations were not significant anymore. The negative association between HDL-c and LGA attenuated to non-significant levels after adjustment for pre-pregnancy BMI and early pregnancy maternal glucose levels. Total cholesterol, LDL-c and non-HDL-c were not associated with LGA. We observed no association between maternal lipid levels in early pregnancy and the risk of SGA.

**Table 4:** Associations of maternal lipid profile in early pregnancy with birth outcomes

	AGA *n* = 4526	SGA OR (95% CI) *n* = 564	***P*** value	LGA OR (95% CI) *n* = 565	***P*** value
**Total cholesterol, SDS**					
Basic model	Reference	0.94 (0.85 to 1.03)	0.16	1.00 (0.92 to 1.10)	0.94
BMI model	Reference	0.96 (0.87 to 1.05)	0.33	0.97 (0.88 to 1.06)	0.51
Glucose model	Reference	0.95 (0.87 to 1.05)	0.32	0.97 (0.87 to 1.07)	0.57
**Triglycerides, SDS**					
Basic model	Reference	0.91 (0.83 to 1.00)	0.04	1.18 (1.07 to 1.29)	0.001
BMI model	Reference	0.94 (0.85 to 1.03)	0.17	1.11 (1.01 to 1.22)	0.04
Glucose model	Reference	0.94 (0.85 to 1.03)	0.19	1.09 (0.99 to 1.20)	0.08
**LDL-c, SDS**					
Basic model	Reference	0.95 (0.87 to 1.04)	0.28	1.01 (0.92 to 1.10)	0.91
BMI model	Reference	0.98 (0.89 to 1.07)	0.59	0.96 (0.88 to 1.06)	0.43
Glucose model	Reference	0.98 (0.89 to 1.07)	0.59	0.97 (0.88 to 1.06)	0.47
**HDL-c, SDS**					
Basic model	Reference	1.01 (0.92 to 1.10)	0.91	0.88 (0.81 to 0.97)	0.01
BMI model	Reference	0.98 (0.90 to 1.07)	0.67	0.92 (0.84 to 1.01)	0.09
Glucose model	Reference	0.98 (0.89 to 1.07)	0.62	0.93 (0.85 to 1.03)	0.16
**Remnant cholesterol, SDS**					
Basic model	Reference	0.91 (0.83 to 1.00)	0.05	1.18 (1.08 to 1.30)	< 0.001
BMI model	Reference	0.94 (0.86 to 1.03)	0.20	1.11 (1.01 to 1.23)	0.03
Glucose model	Reference	0.94 (0.86 to 1.04)	0.23	1.10 (1.00 to 1.21)	0.06
**Non-HDL-c, SDS**					
Basic model	Reference	0.93 (0.85 to 1.02)	0.13	1.05 (0.96 to 1.15)	0.27
BMI model	Reference	0.96 (0.88 to 1.06)	0.40	1.00 (0.91 to 1.09)	0.95
Glucose model	Reference	0.96 (0.88 to 1.06)	0.41	1.00 (0.91 to 1.09)	0.92

*Abbreviations*: *OR* odds ratio, *CI* confidence interval, *SDS* SD scores, *AGA* average-for-gestational age, *SGA* small-for-gestational age, *LGA* large-for-gestational age, *LDL-c* low-density lipoprotein cholesterol, *HDL-c* high-density lipoprotein cholesterol. Values are odds ratio’s (95% CI) derived from multiple logistic regression analyses. Basic model: adjusted for maternal age, gestational age at blood sampling, ethnicity, educational level, parity, smoking and folic acid supplementation. BMI model: basic model additionally adjusted for pre-pregnancy BMI. Glucose model: BMI model additionally adjusted for glucose levels in early pregnancy

In this study, 218 women had gestational hypertension and 139 women had pre-eclampsia in their index pregnancy. Excluding these women from the analysis, it shows that maternal lipid concentrations in early pregnancy are not associated with SGA and LGA (Additional file 1, Table S1).

Additional file 1, Table S2a, presents the lipid profile of 1915 nulliparous, non-smoking women with a BMI < 25 kg/m^2^. This more homogeneous subset of relatively healthy women had a less atherogenic lipid profile in early pregnancy, especially lower triglycerides, LDL-c, remnant cholesterol and higher levels of HDL-c than women in the total study population. In this subset of women, we found no association with adverse birth outcomes (Additional file 1, Table S2b).

## Discussion

This study shows that maternal triglycerides and remnant cholesterol levels in early pregnancy are associated with increased foetal growth rates, specifically for head and abdominal circumference from mid-pregnancy onwards resulting in a higher head circumference at birth and a higher birth weight. Moreover, triglycerides and remnant cholesterol in early pregnancy are associated with a higher risk of being born LGA, independent of maternal BMI. However, these associations attenuated to non-significant levels after adjustment for early pregnancy maternal glucose levels.

The positive association of triglycerides and birth weight that we found is in line with previous studies examining the associations of maternal triglycerides in early, mid- and late pregnancy with birth weight and LGA [41–44]. A study of Vrijkotte et al. examined early pregnancy (median 13 weeks, interquartile range 12; 14 weeks) non-fasting total cholesterol and triglyceride levels in 2502 Dutch women [43]. Lipid levels were divided into quintiles, and women in the highest quintile had a mean triglyceride level of 2.15 (± 0.52) mmol/L. In our study, women had lower levels of triglycerides with a mean of 1.72 (± 0.54) mmol/L in the highest quintile. The study of Vrijkotte et al. found that the highest triglyceride quintile was associated with a higher birth weight and a higher prevalence of a child born LGA. However, a limitation of this study is that they did not take the influence of glucose into account, even though this is a well-known confounder for triglyceride levels [45, 46]. In our study, we corrected for both maternal BMI and early pregnancy glucose levels and showed that the positive association between triglycerides and LGA remained significant if maternal BMI was considered. However, after adjustment for glucose levels, the association attenuated to non-significant levels.

A study by Jin et al. measured lipid levels in 934 women during every trimester and, as expected, found that triglyceride levels increased from 2.2 mmol/L in the first trimester to 3 mmol/L [47]. In this study, higher triglyceride concentrations in late pregnancy were associated with an increased risk of LGA but also a lower risk of SGA. Results were adjusted for some confounders including pre-pregnancy BMI but not glucose. In our study, the expected negative association of early pregnancy maternal lipid levels with SGA was not found. However, it should be noted that the study by Jin et al. used no national representative reference curves and that the reference curves dated from 1989. It is doubtful if these reference curves are still valid as improvements in the past decades, such as the educational level of the mothers and prenatal nutritional status, led to an increase in infant weight [48]. It may be possible that this has led to an underestimation of SGA (2.4%) and an overestimation of LGA (26.3%). Maternal triglycerides have also been positively associated with newborn fat which may be a more accurate measure of newborn adiposity than birth weight [17, 18]. Triglycerides and remnant cholesterol levels reflect an impaired metabolism of triglyceride-rich lipoproteins and their remnants, which are controlled by placental lipoprotein lipases such as placental lipoprotein lipase (pLPL) and placental endothelial lipase (pEPL) activity [13, 14].

The only study which assessed maternal remnant cholesterol levels, representative of the remnant lipoproteins, was performed in mice and showed that remnant cholesterol is associated with accelerated foetal growth in mice [49]. In our study, maternal remnant cholesterol levels in early pregnancy are positively associated with foetal growth, increased foetal growth pattern of head circumference and abdominal circumference and the risk of LGA, independent of maternal BMI. After correction for glucose, the association of remnant cholesterol and LGA attenuated to just non-significance. This may be explained by the close relation between remnant cholesterol levels and insulin resistance, the association of insulin resistance with glucose levels and the association of insulin resistance with an atherogenic plasma lipid profile [50–52].

To fully comprehend the association of maternal lipid levels and foetal growth, it is important to understand the maternal-placental-foetal transport pathways but also the development of the foetal lipid metabolism. Unfortunately, to date, this is still largely unknown. However, we assume that the contribution of the foetal metabolism will be ignorable or very little in early pregnancy, and therefore, we expect that this will have a limited effect on our results. Our results are in line with a meta-analysis of Wang et al. describing a positive association of triglycerides with LGA and birth weight and a negative association of HDL-c with birth weight [53]. The associations were even stronger in overweight or obese women prior to pregnancy. Our study adds to these findings that the associations are even independent of pre-pregnancy BMI.

It is known that children of mothers with high cholesterol levels in early pregnancy have a higher risk of fatty streaks in the aorta [54]. These fatty streaks are a precursor of atherosclerosis and can result in cardiovascular disease later in life [55]. These results underline the importance of measuring maternal lipid levels in early pregnancy.

It has been suggested that maternal fasting glucose levels, postpandrial glucose levels and non-fasting random samples are appropriate measures of maternal glucose metabolism and are related to adverse birth outcomes [56, 57]. A recent study of Barbour et al. suggests that postpandrial triglyceride levels may be a new target for early intervention in obese pregnancies [17], since postpandrial triglycerides compared to fasting triglycerides are better predictors of newborn adiposity. The findings of our study are independent of maternal BMI, and non-fasting blood values may better reflect the normal physiological state in pregnant women [5, 58]. Therefore, we suggest that further studies are needed to replicate our findings, including fasting blood samples and detailed postpandrial measurements.

A few studies examining the effect of HDL-c levels and the risk of LGA did not observe an association [47, 59]. We hypothesized that HDL-c was in contrast to the other lipid levels negatively associated with foetal growth. However, the negative associations of HDL-c with birth weight and LGA attenuated to non-significance after adjustment for pre-pregnancy BMI and glucose. This may be explained by the inverse association between BMI and HDL-c [60].

In this study, we found no association of lipid levels in early pregnancy with adverse birth outcomes in a subset of relatively healthy women (nulliparous, non-smoking, lean women). If the association of lipid levels with adverse birth outcomes would be fully explained by genetics, we would have expected to also find an association of early pregnancy lipid levels with adverse birth outcomes in this relatively healthy population. Since no association was found, we hypothesize that in addition to genetics, lifestyle factors also play an important role in the association of lipid levels with adverse birth outcomes.

Currently, non-high-density lipoprotein cholesterol (non-HDL-c) is often used to describe the total of pro-atherogenic particles (low-density lipoprotein cholesterol [LDL-c], lipoprotein-a, intermediate-density lipoprotein [IDL] and VLDL). High maternal levels of non-HDL-c may therefore be more specific to depict the future cardiovascular risk of the foetus than maternal hypercholesterolemia [61]. This study is to our knowledge the first study to examine the role of maternal early pregnancy lipids including remnant cholesterol and non-HDL-c in association with LGA independent of maternal pre-pregnancy BMI and glucose levels.

Our results suggest a novel association of early pregnancy maternal lipid levels and the risk of a child born LGA. However, it should be noted that foetal growth alone may be a weak surrogate for perinatal harm since there was no difference in NICU admission for women with a child born AGA compared to women with a child born LGA in our study. Before implementation of lifestyle interventions to decrease maternal lipid levels in early pregnancy, future studies are warranted to examine whether maternal lipid levels are not only associated with foetal growth, but also with subsequent perinatal harm such as shoulder dystocia, neonatal asphyxia and neonatal death.

### Strengths and limitations

Blood samples were obtained in a non-fasting state, sampled on nonfixed times throughout the day. This may have led to non-differential misclassification, causing an underestimation of the observed effect estimates. However, several large-scale, population-based studies and registries including children, women, men and patients with diabetes have established that plasma lipids and lipoproteins change only modestly in response to habitual food intake [62–67]. Among all studies comparing non-fasting with fasting lipid profiles, minor increases in plasma triglycerides and minor decreases in total and LDL cholesterol concentrations were observed, with no change in HDL cholesterol concentrations [67]. These minor and transient changes in lipid concentrations appear to be clinically insignificant [67]. Naturally, the corresponding changes in concentrations in individuals will differ from mean changes in populations, exactly as concentrations will differ from one fasting measurement to another in the same individual [67]. Lipids and lipoproteins change minimally in response to normal food intake [67, 68]. It is stated that only if non-fasting plasma triglycerides are > 5 mmol/L, a fasting blood sample could be considered [67]. In our study, in only three women, triglycerides exceeded 5 mmol/L. Moreover, using non-fasting samples may also have advantages. As previously mentioned, they may better reflect the normal physiological state in pregnant women. Moreover, non-fasting triglycerides were previously observed to be better predictors of newborn adiposity [17]. Compared to non-responders, study participants were more often of European origin, were higher educated, had a lower pre-pregnancy BMI, were more often nulliparous, smoked more often in their pregnancy and more often used folic acid supplementation (Additional file 1, Table S3). It is not possible to foresee whether and, if so, how this would affect our results. Moreover, we included a relatively healthy population and blood samples were obtained in early pregnancy whilst dyslipidaemia is more profound in mid- and late pregnancy which both may have led to an underestimation of the association between maternal lipid profile in early pregnancy with foetal growth and the risk of LGA [69]. Therefore, our results cannot be extrapolated beyond this context. Pre-pregnancy BMI was calculated from self-reported pre-pregnancy weight. However, pre-pregnancy BMI was strongly correlated with BMI measured at enrolment, which makes misclassification unlikely. Gestational weight gain may also have an effect on our results; however, due to the high percentage of missing information (59.3%) and its occurrence after our exposure, we did not include gestational weight gain to our models. In future studies, the role of gestational weight gain should be further examined.

## Conclusion

This study suggests a novel association of triglycerides and remnant cholesterol in early pregnancy with foetal growth rates and the risk of a child born LGA. However, future studies are warranted to explore clinical implication possibilities.

## Supplementary information

**Additional file 1: Figure S1.** Directed acyclic graph (DAG) of exposure, outcome and possible covariates. **Table S1.** Association of maternal lipid profile in early pregnancy with birth outcomes, excluding women with a hypertensive disorder of pregnancy. **Table S2a.** Maternal lipid profile in early pregnancy of the total population (*n* = 5702) and a subgroup of nulliparous, non-smoking women with a pre-pregnancy BMI < 25 kg/m2 (*n* = 1915). **Table S2b.** Association of maternal lipid profile in early pregnancy with birth outcomes in a subgroup of nulliparous, non-smoking women with a pre-pregnancy BMI < 25 kg/m2 (n = 1915). **Table S3.** Baseline characteristics between women with and without available lipid measurements in early pregnancy.

**Additional file 2.** STROBE statement.

## Data Availability

Data requests can be made to the secretariat of Generation R.

## Abbreviations

AGA: Average-for-gestational age
ApoE: Apolipoprotein E
BMI: Body mass index
CI: Confidence interval
DAG: Directed acyclic graph
DOHaD: Developmental Origins of Health and Disease
EFW: Estimated foetal weight
FFA: Free fatty acids
HDL-c: High-density lipoprotein cholesterol
IDL: Intermediate-density lipoprotein
LDL-c: Low-density lipoprotein cholesterol
LGA: Large-for-gestational age
LPL: Lipoprotein lipase
NICU: Neonatal Intensive Care Unit
OR: Odds ratio
pEPL: Placental endothelial lipase
pLPL: Placental lipoprotein lipase
SD: Standard deviation
SDS: Standard deviation scores
SGA: Small-for-gestational age
VLDL: Very low-density lipoprotein
