# Supplementary material for: Maternal lipid profile in early pregnancy is associated with foetal growth and the risk of a child born large-for-gestational age: a population-based prospective cohort study: Maternal lipid profile in early pregnancy and foetal growth
Source: BMC Med. 2020 Oct 2;18:276. doi: 10.1186/s12916-020-01730-7 (PMC7532083; doi:10.1186/s12916-020-01730-7)
Supplement: Supplementary file 1 — Additional file 1: Figure S1. Directed acyclic graph (DAG) of exposure, outcome and possible covariates. Table S1. Association of maternal lipid profile in early pregnancy with birth outcomes, excluding women with a hypertensive disorder of pregnancy. Table S2a. Maternal lipid profile in early pregnancy of the total population (n = 5702) and a subgroup of nulliparous, non-smoking women with a pre-pregnancy BMI < 25 kg/m2 (n = 1915). Table S2b. Association of maternal lipid profile in early pregnancy with birth outcomes in a subgroup of nulliparous, non-smoking women with a pre-pregnancy BMI < 25 kg/m2 (n = 1915). Table S3. Baseline characteristics between women with and without available lipid measurements in early pregnancy. [file 12916_2020_1730_MOESM1_ESM.docx]

**SUPPLEMENTAL MATERIAL**

**Additional file 1, Figure S1.**

**
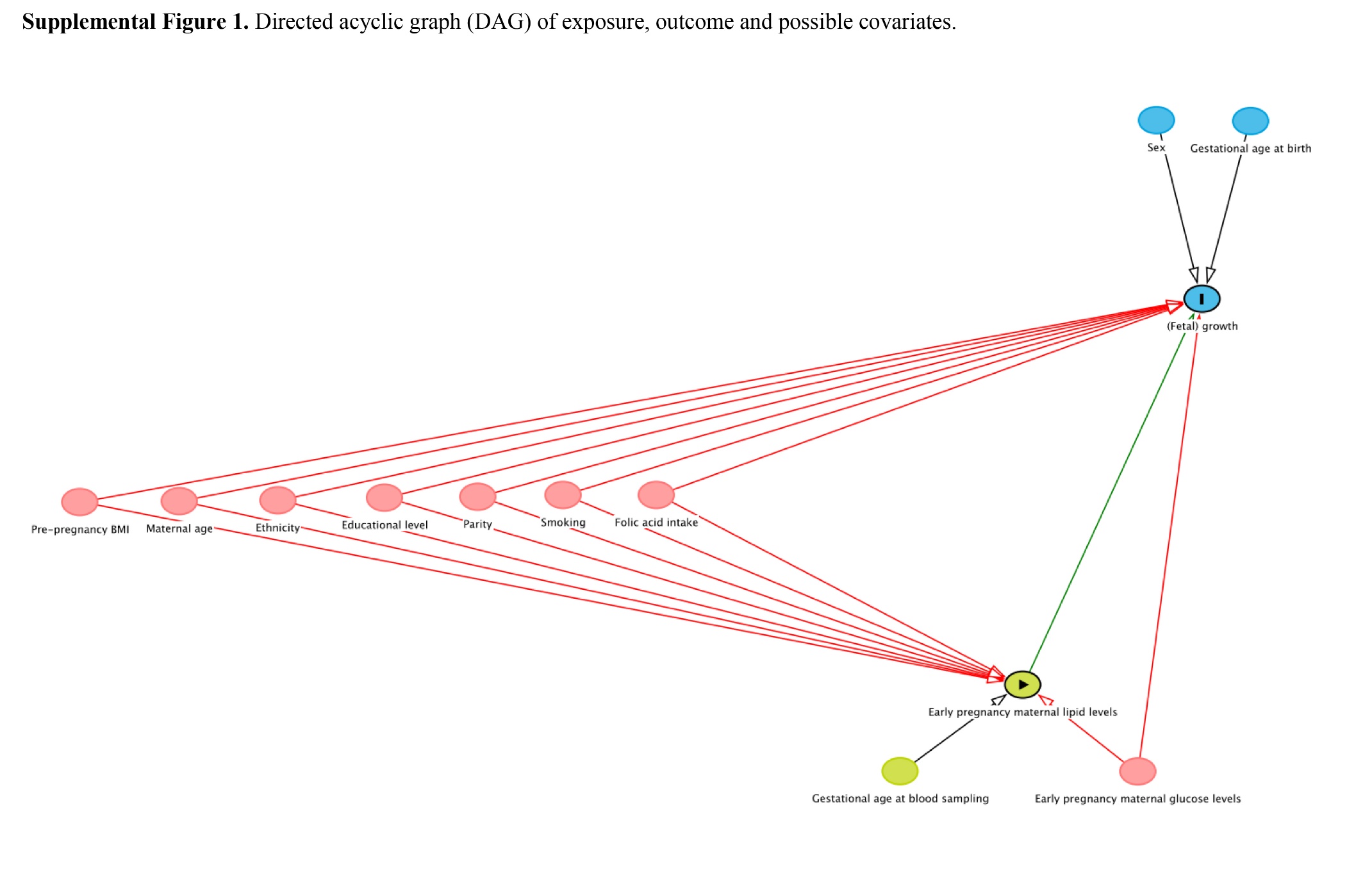
**

**Additional File 1, Table S1.** Associations of maternal lipid profile in early pregnancy with birth outcomes, excluding women with a hypertensive disorder of pregnancy.

|  | **AGA**  n = 4280 | **SGA**  OR (95% CI)  n = 496 | ***P*-value** | **LGA**  OR (95% CI)  n = 526 | ***P*-value** |
| --- | --- | --- | --- | --- | --- |
| **Total-cholesterol, SDS** |  |  |  |  |  |
| Basic model | Reference | 0.94 (0.85 to 1.04) | 0.21 | 1.01 (0.92 to 1.11) | 0.87 |
| BMI model | Reference | 0.96 (0.87 to 1.06) | 0.41 | 0.98 (0.89 to 1.07) | 0.60 |
| Glucose model | Reference | 0.96 (0.87 to 1.06) | 0.40 | 0.98 (0.89 to 1.08) | 0.68 |
| **Triglycerides, SDS** |  |  |  |  |  |
| Basic model | Reference | 0.90 (0.82 to 0.99) | 0.03 | 1.16 (1.05 to 1.27) | 0.003 |
| BMI model | Reference | 0.93 (0.84 to 1.03) | 0.15 | 1.09 (0.99 to 1.20) | 0.09 |
| Glucose model | Reference | 0.93 (0.84 to 1.03) | 0.16 | 1.07 (0.97 to 1.18) | 0.18 |
| **LDL-c, SDS** |  |  |  |  |  |
| Basic model | Reference | 0.96 (0.87 to 1.06) | 0.40 | 1.01 (0.92 to 1.11) | 0.91 |
| BMI model | Reference | 0.99 (0.89 to 1.09) | 0.76 | 0.97 (0.88 to 1.06) | 0.49 |
| Glucose model | Reference | 0.98 (0.89 to 1.09) | 0.75 | 0.97 (0.88 to 1.07) | 0.54 |
| **HDL-c, SDS** |  |  |  |  |  |
| Basic model | Reference | 1.00 (0.91 to 1.11) | 0.94 | 0.90 (0.82 to 0.99) | 0.03 |
| BMI model | Reference | 0.98 (0.89 to 1.08) | 0.65 | 0.94 (0.85 to 1.03) | 0.18 |
| Glucose model | Reference | 0.98 (0.88 to 1.08) | 0.61 | 0.95 (0.86 to 1.05) | 0.31 |
| **Remnant cholesterol, SDS** |  |  |  |  |  |
| Basic model | Reference | 0.90 (0.82 to 1.00) | 0.04 | 1.17 (1.06 to 1.28) | 0.002 |
| BMI model | Reference | 0.93 (0.85 to 1.03) | 0.17 | 1.10 (1.00 to 1.21) | 0.06 |
| Glucose model | Reference | 0.94 (0.85 to 1.03) | 0.19 | 1.08 (0.98 to 1.19) | 0.13 |
| **Non-HDL-c, SDS** |  |  |  |  |  |
| Basic model | Reference | 0.94 (0.85 to 1.03) | 0.19 | 1.05 (0.96 to 1.15) | 0.32 |
| BMI model | Reference | 0.97 (0.88 to 1.07) | 0.51 | 1.00 (0.90 to 1.10) | 0.94 |
| Glucose model | Reference | 0.97 (0.88 to 1.07) | 0.51 | 1.00 (0.90 to 1.10) | 0.92 |

Abbreviations: OR, odds ratio; CI, confidence interval; SDS, SD-scores; AGA, average-for-gestational age; SGA, small-for-gestational age; LGA, large-for-gestational age; LDL-c, low-density lipoprotein cholesterol; HDL-c, high-density lipoprotein cholesterol. Values are odds ratio’s (95% CI) derived from multiple logistic regressions analyses. Basic model: adjusted for maternal age, gestational age at blood sampling, ethnicity, educational level, parity, smoking and folic acid supplementation. BMI model: basic model additionally adjusted for pre-pregnancy BMI. Glucose model: BMI model additionally adjusted for glucose levels in early pregnancy.

**Additional file 1, Table S2a.** Maternal lipid profile in early pregnancy of the total population (n = 5702) and of a subgroup of nulliparous, non-smoking women with a pre-pregnancy BMI < 25 kg/m2 (n = 1915).

|  | Total population  (n = 5702) | Nulliparous, non-smoking,  with BMI < 25 kg/m2  (n = 1915) |
| --- | --- | --- |
| Total cholesterol, mmol/L | 4.82 (0.87) | 4.80 (0.86) |
| Triglycerides, mmol/L | 1.27 (0.72; 2.36) | 1.18 (0.69; 2.07) |
| LDL-c, mmol/L | 2.43 (0.72) | 2.39 (0.70) |
| HDL-c, mmol/L | 1.77 (0.35) | 1.84 (0.34) |
| Remnant cholesterol, mmol/L | 0.62 (0.33; 1.07) | 0.54 (0.31; 0.94) |
| Non-HDL-c, mmol/L | 3.05 (0.83) | 2.96 (0.80) |

Abbreviations: LDL-c, low-density lipoprotein cholesterol; HDL-c, high-density lipoprotein cholesterol; BMI, Body Mass Index. Data are presented as mean (SD) for continuous variables with a normal distribution, or as median (90% range) for continuous variables with a skewed distribution.

**Additional file 1, Table S2b.** Associations of maternal lipid profile in early pregnancy with birth outcomes in a subgroup of nulliparous, non-smoking women with a pre-pregnancy BMI < 25 kg/m2 (n = 1915).

|  | **AGA**  n = 1550 | **SGA**  OR (95% CI)  n = 210 | ***P*-value** | **LGA**  OR (95% CI)  n = 142 | ***P*-value** |
| --- | --- | --- | --- | --- | --- |
| **Total-cholesterol, SDS** |  |  |  |  |  |
| Basic model | Reference | 1.08 (0.92 to 1.26) | 0.36 | 1.03 (0.86 to 1.25) | 0.73 |
| Glucose model | Reference | 1.08 (0.92 to 1.26) | 0.36 | 1.04 (0.86 to 1.25) | 0.72 |
| **Triglycerides, SDS** |  |  |  |  |  |
| Basic model | Reference | 0.96 (0.81 to 1.12) | 0.58 | 1.06 (0.87 to 1.30) | 0.54 |
| Glucose model | Reference | 0.96 (0.81 to 1.13) | 0.60 | 1.06 (0.87 to 1.30) | 0.56 |
| **LDL-c, SDS** |  |  |  |  |  |
| Basic model | Reference | 1.07 (0.91 to 1.25) | 0.40 | 1.07 (0.88 to 1.29) | 0.51 |
| Glucose model | Reference | 1.07 (0.91 to 1.26) | 0.40 | 1.07 (0.88 to 1.29) | 0.51 |
| **HDL-c, SDS** |  |  |  |  |  |
| Basic model | Reference | 1.07 (0.92 to 1.26) | 0.37 | 0.93 (0.77 to 1.12) | 0.43 |
| Glucose model | Reference | 1.07 (0.91 to 1.26) | 0.40 | 0.93 (0.77 to 1.13) | 0.45 |
| **Remnant cholesterol, SDS** |  |  |  |  |  |
| Basic model | Reference | 0.96 (0.81 to 1.12) | 0.58 | 1.06 (0.87 to 1.29) | 0.54 |
| Glucose model | Reference | 0.96 (0.82 to 1.13) | 0.60 | 1.06 (0.87 to 1.29) | 0.56 |
| **Non-HDL-c, SDS** |  |  |  |  |  |
| Basic model | Reference | 1.05 (0.89 to 1.23) | 0.57 | 1.07 (0.88 to 1.30) | 0.48 |
| Glucose model | Reference | 1.05 (0.89 to 1.23) | 0.56 | 1.07 (0.88 to 1.30) | 0.49 |

Abbreviations: OR, odds ratio; CI, confidence interval; SDS, SD-scores; AGA, average-for-gestational age; SGA, small-for-gestational age; LGA, large-for-gestational age; LDL-c, low-density lipoprotein cholesterol; HDL-c, high-density lipoprotein cholesterol; BMI, Body Mass Index. Values are odds ratio’s (95% CI) derived from multiple logistic regressions analyses. Basic model: adjusted for maternal age, gestational age at blood sampling, ethnicity, educational level, and folic acid supplementation. Glucose model: basic model additionally adjusted for glucose levels in early pregnancy.

**Additional file 1, Table S3.** Baseline characteristics between women with and without available lipid measurements in early pregnancy

|  | **Lipid measurements available**  n = 5702 | **No lipid measurements available**  n = 2151 | ***P*-value** |
| --- | --- | --- | --- |
| **Maternal characteristics** |  |  |  |
| Maternal age at enrolment (years) | 29.5 (5.1) | 29.5 (6.0) | 0.68 |
| Non-European ethnicity, n (%) | 2185 (40.0) | 1077 (55.9) | <0.001 |
| Educational level |  |  | <0.001 |
| Primary or no education, n(%) | 535 (10.1) | 324 (17.8) |  |
| Secondary, n (%) | 2448 (46.4) | 910 (49.9) |  |
| Higher, n (%) | 2296 (43.5) | 591 (32.4) |  |
| Pre-pregnancy BMI (kg/m^2^) |  |  | 0.02 |
| Normal or underweight (<25.0), n (%) | 3418 (72.8) | 1137 (70.3) |  |
| Overweight (25.0-30.0), n (%) | 901 (19.2) | 314 (19.4) |  |
| Obesity (≥ 30.0), n (%) | 377 (8.0) | 167 (10.3) |  |
| Nulliparous, n (%) | 3484 (61.7) | 1037 (49.5) | <0.001 |
| Smoking during pregnancy, n (%) | 1474 (28.9) | 428 (24.2) | <0.001 |
| No folic acid supplementation, n (%) | 1125 (25.7) | 624 (43.3) | <0.001 |
| **Birth characteristics** |  |  |  |
| Gestational age at birth (weeks) | 40.1 (36.9; 42.1) | 40.0 (36.3; 42.1) | <0.001 |
| Boy, n (%) | 2880 (50.5) | 1071 (50.5) | 0.98 |
| Birth weight, (grams) | 3401 (560) | 3377 (570) | 0.10 |
| SGA, n (%) | 564 (11.1) | 190 (9.1) | 0.24 |
| LGA, n (%) | 565 (11.1) | 207 (10.9) | 0.95 |

Abbreviations: BMI, Body Mass Index; SGA, small-for-gestational age; LGA, large-for-gestational age. Data are presented as valid percentages for categorical variables, mean (SD) for continuous variables with a normal distribution, or as median (90% range) for continuous variables with a skewed distribution. Presented values are not imputed.
